# Targeting the SUMO pathway as a novel treatment for anaplastic thyroid cancer

**DOI:** 10.18632/oncotarget.21954

**Published:** 2017-10-23

**Authors:** James P. De Andrade, Allison W. Lorenzen, Vincent T. Wu, Maria V. Bogachek, Jung M. Park, Vivian W. Gu, Claire M. Sevenich, Victoria C. Cassady, Anna C. Beck, Mikhail V. Kulak, Robert A. Robinson, Geeta Lal, Ronald J. Weigel

**Affiliations:** ^1^ Department of Surgery, University of Iowa, Iowa City, IA, USA; ^2^ Department of Anatomy and Cell Biology, University of Iowa, Iowa City, IA, USA; ^3^ Department of Molecular Physiology and Biophysics, University of Iowa, Iowa City, IA, USA; ^4^ Department of Pathology, University of Iowa, Iowa City, IA, USA

**Keywords:** SUMO, sumoylation, TFAP2A, anaplastic thyroid cancer

## Abstract

Cancer stem cells (CSCs) are expanded in anaplastic thyroid cancer (ATC) and standard treatment approaches have failed to improve survival, suggesting a need to specifically target the CSC population. Recent studies in breast and colorectal cancer demonstrated that inhibition of the SUMO pathway repressed CD44 and cleared the CSC population, mediated through SUMO-unconjugated TFAP2A. We sought to evaluate effects of inhibiting the SUMO pathway in ATC. ATC cell lines and primary ATC tumor samples were evaluated. The SUMO pathway was inhibited by knockdown of PIAS1 and use of SUMO inhibitors anacardic acid and PYR-41. The expression of TFAP2A in primary ATC was examined by immunohistochemistry. All ATC cell lines expressed TFAP2A but only 8505C expressed SUMO-conjugated TFAP2A. In 8505C only, inhibition of the SUMO pathway by knockdown of PIAS1 or treatment with SUMO inhibitors repressed expression of CD44 with a concomitant loss of SUMO-conjugated TFAP2A. The effect of SUMO inhibition on CD44 expression was dependent upon TFAP2A. Treatment with SUMO inhibitors resulted in a statistically improved tumor-free survival in mice harboring 8505C xenografts. An examination of primary ATC tissue determined that TFAP2A was expressed in 4 of 11 tumors surveyed. We conclude that inhibition of the SUMO pathway repressed the CSC population, delaying the outgrowth of tumor xenografts in ATC. The effect of SUMO inhibition was dependent upon expression of SUMO-conjugated TFAP2A, which may serve as a molecular marker for therapeutic effects of SUMO inhibitors. The findings provide pre-clinical evidence for development of SUMO inhibitors for the treatment of ATC.

## INTRODUCTION

It has been proposed that all cancers share a set of six physiologic alterations: autonomous growth signaling, resistance to growth inhibitory signals, circumvention of apoptosis, immortalization, angiogenesis, and the potential for invasion and metastasis [1]. With a greater understanding of the biology of cancer, targeted therapy for a variety of malignancies heralds an era of cancer treatment based on greater predictability of effectiveness, lower risks of complications, and lower costs. However, the current reality is that targeted cancer therapy is limited to a minority of cancers, can have significant toxicity, is associated with high costs, and is often accompanied by the development of drug-resistant recurrences [2]. Hence, it has been proposed that future development of novel cancer therapies should focus on low-toxicity, “broad-spectrum” approaches that target key pathways common to cancers [2].

The concept of a cancer stem cell (CSC) population has gained greater acceptance in a variety of malignancies. Models of breast cancer have proved to be a valuable resource to develop paradigms for the biology of CSCs in solid tumors. The CSCs or tumor-initiating cells (TICs) are postulated to result from oncogenic mutations occurring in normal tissue stem cells [3–5]. CSCs have the capacity for self-renewal and can also give rise to the heterogeneous tumor cell population, which include the more differentiated tumor cells. Clinically, it is hypothesized that the CSC population is, largely responsible for cancer recurrence, metastasis and resistance to chemotherapy; hence, durable treatments for cancer require effective elimination of the CSC population to prevent cancer recurrence.

The process of epithelial-mesenchymal transition (EMT) is closely linked to the acquisition of stem cell properties [6]. As cancer cells undergo EMT, they lose expression of epithelial differentiation markers and gain expression of genes such as CD44, which is a key marker for CSCs. EMT is regulated by a number of transcription factors and, interestingly, many of the transcriptional regulators of EMT are controlled by SUMO conjugation [7]. Sumoylation is a type of post-translational modification involving the covalent attachment of small ubiquitin-like modifier (SUMO) proteins to target proteins, a number of which are thought to play a role in oncogenesis and cancer progression [8]. Recent evidence indicates that the AP-2 transcription factor family plays an important role in EMT and maintenance of the CSC subpopulation. Loss of the TFAP2C transcription factor induced EMT in luminal breast cancers [9]. We previously showed that the highly homologous factor, TFAP2A, normally lacks transcriptional activity at luminal gene promoters due to SUMO conjugation [10]. However, inhibiting the SUMO pathway induced TFAP2A activity that repressed CD44 expression and induced other changes in gene expression consistent with mesenchymal-epithelial transition (MET). Similarly, inhibiting the SUMO pathway repressed the outgrowth of basal breast and colon cancer xenografts, suggesting that SUMO inhibitors might offer a broad-based approach to cancer therapy [10–13].

To explore the approach of developing SUMO inhibitors as a broad therapeutic strategy in cancer, it is important to determine the cancer types that might be responsive to targeting the SUMO pathway. Based on earlier experience, aggressive cancer types with an expanded CSC population and an EMT signature would be more likely to be sensitive to SUMO inhibitors. Anaplastic thyroid cancers (ATC) have an expanded EMT/CSC population compared to well-differentiated thyroid cancer [14]. Furthermore, ATC has a dismal prognosis with limited therapeutic options. Hence, there is a pressing need to investigate alternative treatment strategies for ATC and therapies that eliminate the thyroid CSC population may result in a more durable response to chemotherapy [15]. Herein, we examined the response of a panel of ATC cell lines to SUMO inhibition and investigated the role of SUMO unconjugated TFAP2A in mediating repression of CD44 expression and altering the CSC/TIC population.

## RESULTS

### Expression of SUMO-conjugated TFAP2A in anaplastic thyroid cancer cell lines

**Figure 1:**
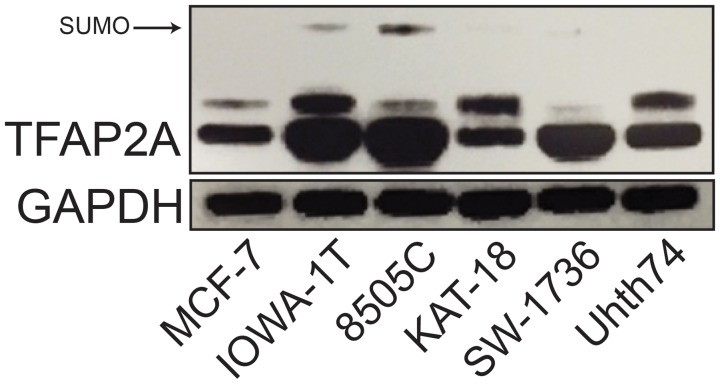
Expression of SUMO-conjugated TFAP2A in ATC Cell Lines Western blot analysis was performed on a panel of breast and ATC cell lines noted. Of the ATC cell lines, western blot for TFAP2A demonstrated SUMO-conjugated TFAP2A only in 8505C.

Inhibition of the SUMO pathway in basal breast and colorectal cancers repressed CD44 expression, which was dependent upon TFAP2A [10, 13]. The findings support the hypothesis that the response to SUMO inhibition may be related to the expression of SUMO-conjugated TFAP2A. To test this hypothesis in ATC, we screened a panel of ATC cell lines for expression of TFAP2A compared to breast cancer cells as a control. As seen in Figure 1, SUMO-conjugated TFAP2A was highly expressed in the basal breast cancer cell line IOWA-1T, whereas only the SUMO-unconjugated form of TFAP2A was detected in the luminal cell line MCF-7. All of the ATC cell lines expressed TFAP2A. However, KAT-18, SW-1736 and Uhth74 had no evidence for SUMO-conjugated TFAP2A. Whereas, 8505C and SW-1736 expressed similar levels of TFAP2A, only 8505C had detectible levels of SUMO-conjugated TFAP2A with levels that were similar to IOWA-1T.

**Figure 2:**
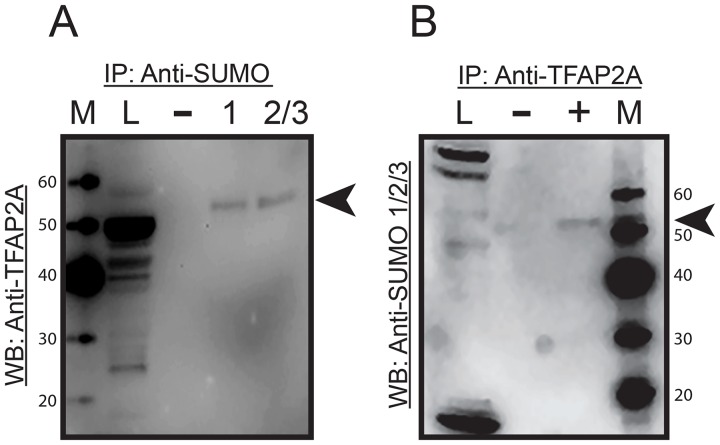
Immunoprecipitation of SUMO Conjugated TFAP2A Protein extracts from 8505C cells was subjected to IP by anti-SUMO-1/2/3 antibodies **(A)** or anti-TFAP2A antibody **(B)** with IP performed with antibodies indicated by (+) or IgG (−) and resolved on western blot with anti-TFAP2A antibody (A) or anti-SUMO-1/2/3 antibodies (B) Load (L) of extract is shown in the first lane and markers (M) in the corresponding lane with size markers noted. In both approaches, SUMO conjugated TFAP2A (arrowhead) was identified as an approximately 55kD protein and was approximately 10 kD larger than SUMO unconjugated TFAP2A. Of note the SUMO-conjugated TFAP2A ran slightly faster in the IP lanes compared to load, and this effect is likely due to the tremendous increased total amount of protein in the load lane; however, degradation during the IP procedure cannot be completely excluded. Unconjugated TFAP2A was note in load lane in panel A and free SUMO was seen at approximately 12kD at the bottom of the load lane in panel B.

To confirm the expression of SUMO-conjugated TFAP2A in 8505C, protein extracts were subjected to immunoprecipitation (IP) with anti-SUMO antibody and resolved on western blot with anti-TFAP2A antibody. As seen in Figure 2A, SUMO-conjugated TFAP2A was identified as a protein of approximately 55kD and is consistent with findings previously reported in breast and colon cancer cell lines [10, 13]. The data also demonstrate that TFAP2A is sumoylated by SUMO-1 and SUMO-2/3. In a parallel experiment, 8505C protein extract was immunoprecipitated with anti-TFAP2A and probed on western blot with anti-SUMO-1/2/3 antibodies (Figure 2B). An identical protein of 55kD is again found, confirming the identity of SUMO-conjugated TFAP2A in 8505C cells.

### Inhibiting the SUMO pathway decreased CD44 expression

**Figure 3:**
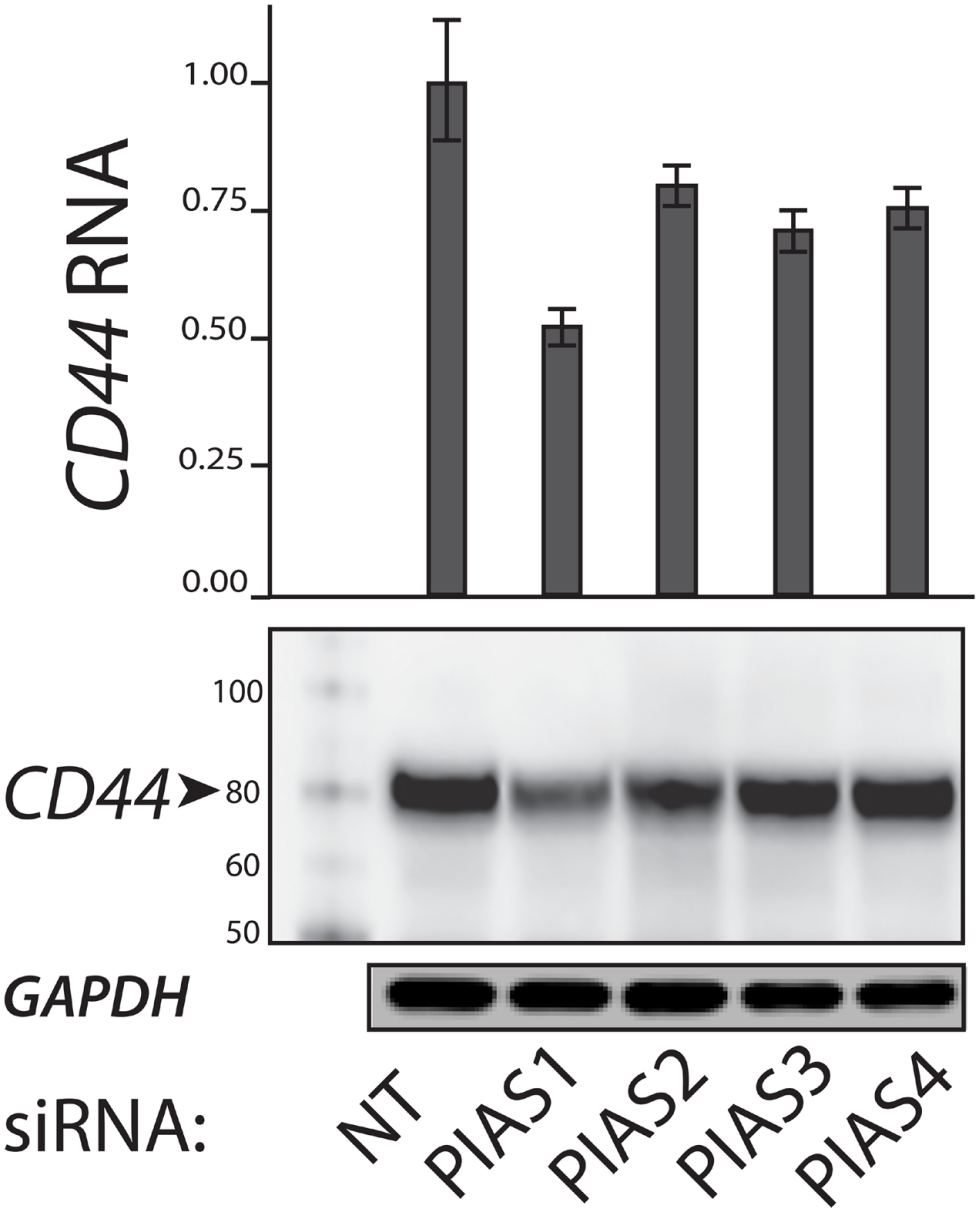
Expression of CD44 with Knockdown of PIAS1-4 Proteins PIAS1-4 were knocked down with siRNA compared to non-targeting (NT) siRNA. Top panel shows relative CD44 mRNA expression normalized to NT. Bottom panels show western blots for CD44 and GAPDH. First lane shows protein markers. Note that knockdown of each PIAS1-4 reduced CD44 expression with the largest effect (by RNA and protein) noted with knockdown of PIAS1.

**Figure 4:**
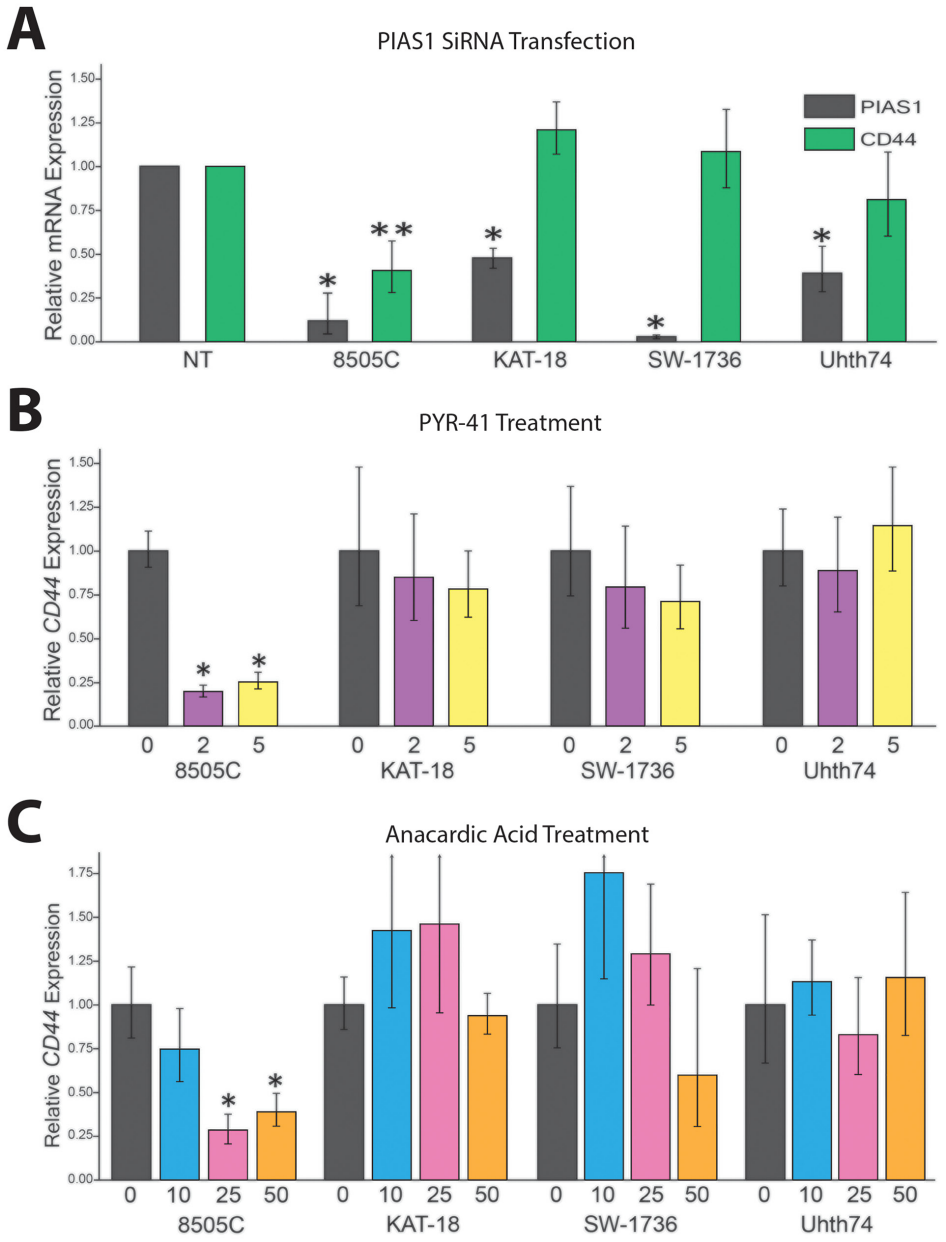
Response of CD44 to SUMO Inhibition in Panel of ATC The panel of ATC cell lines was screened for the effect of SUMO inhibition on CD44 RNA expression, determined by RT-PCR. **(A)** Cell lines indicated were transfected with PIAS1 vs. non-targeting (NT) siRNA and examined for effect on CD44 expression; only 8505C demonstrated significant reduction of CD44 expression. **(B/C)** The panel of ATC cell lines was tested for a dose-dependent effect on CD44 expression with treatment with PYR-41 with vehicle only (0), 2 μM (2) or 5 μM (5) (panel B) or anacardic acid with vehicle (0), 10 μM (10), 25 μM (25) or 50 μM (50) (panel C) with treatment for 72 hours. Response to SUMO inhibitors was only noted in 8505C.

We sought to determine the effect of SUMO inhibition on CD44 expression in ATC cell lines and to establish a correlation with SUMO-conjugated TFAP2A. In mammals there are four genes in the family of Protein Inhibitor of Activated STAT (PIAS) SUMO E3 ligases—*PIAS1, PIAS2* (*PIASX*), *PIAS3*, and *PIAS4* (*PIASγ*) [16]. Our earlier work in breast and colon cancers found that knockdown of PIAS1 repressed CD44 expression. To determine the effect of the different PIAS proteins in ATC, we screened the ability for knockdown of the SUMO E3 ligases PIAS1-4 to alter expression of CD44 in 8505C cells. Knockdown of PIAS1 had the greatest effect in altering CD44 expression (Figure 3). Hence, further experiments focused on the PIAS1 E3 ligase. PIAS1 was knocked down with siRNA in the panel of ATC cell lines and expression of CD44 was assessed. Successful knockdown of PIAS1 protein was confirmed by western blot (see Supporting Information, Supplementary Figure 1 Figure 1.). Consistent with data in Figure 3, knockdown of PIAS1 in 8505C significantly repressed expression of CD44 (Figure 4A); however, knockdown of the SUMO pathway enzyme had no effect on CD44 expression in KAT-18, SW-1736, and Uhth74. The panel of ATC cell lines was treated with PYR-41 and anacardic acid (AA), both compounds known to be small molecule SUMO inhibitors (Figures 4B & 4C). PYR-41 treatment resulted in statistically significant reduction in CD44 expression in 8505C at both 2 μM and 5 μM (77% reduction p < 0.05, and 74% reduction p < 0.05, respectively). By comparison, PYR-41 treatment had no effect on CD44 expression in KAT-18, SW-1736, and Uhth74. Similarly, AA treatment at 25 μM and 50 μM repressed CD44 expression in 8505C cells (73% reduction, p <0.05, 63% reduction, p < 0.05, respectively). Consistent with results using PYR-41, AA had no effect on CD44 expression in KAT-18, SW-1736 and Uhth74 at any concentration. Interestingly, the effects of SUMO inhibition on CD44 expression in the panel of ATC cell lines demonstrated a correlation with expression of SUMO-conjugated TFAP2A.

**Figure 5:**
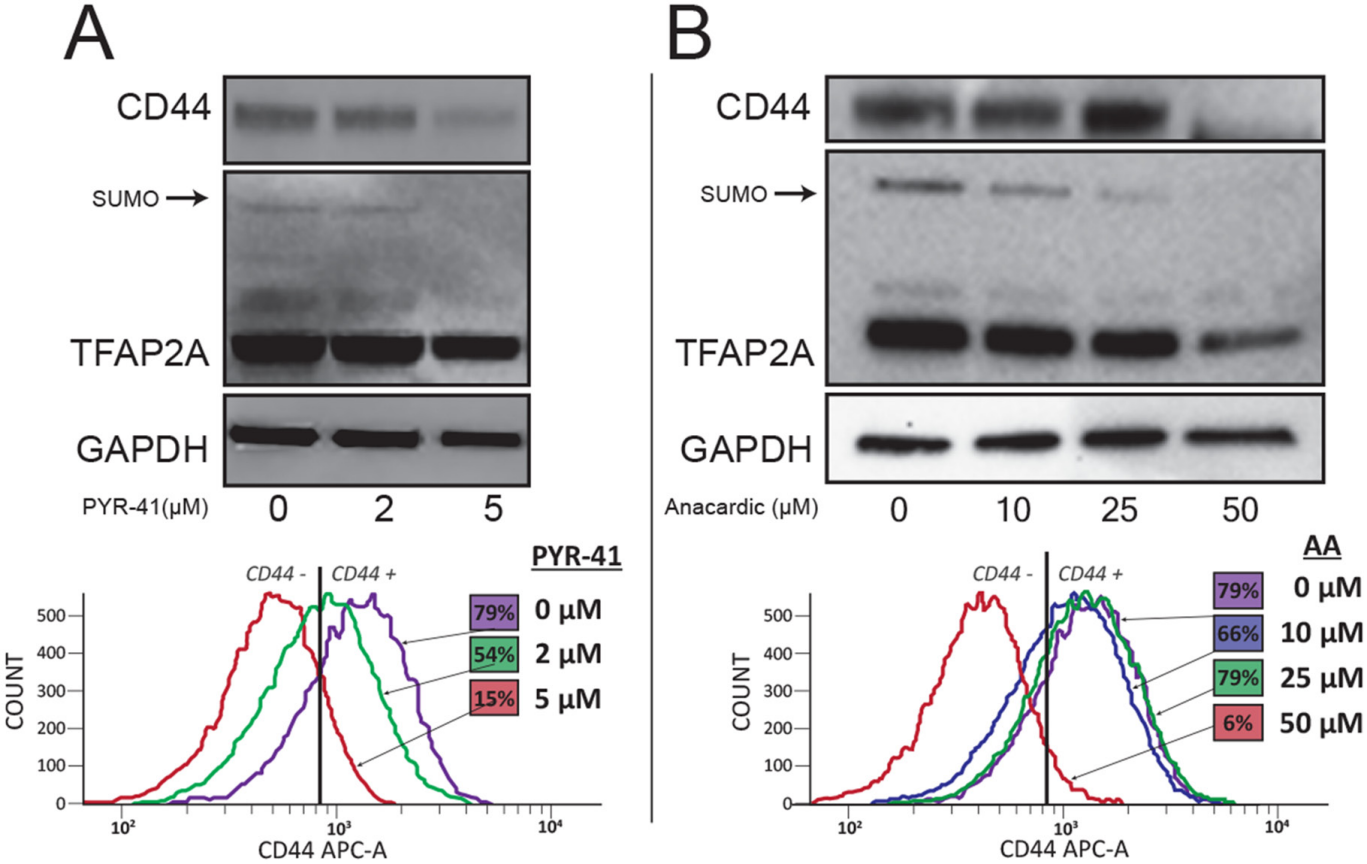
Repression of CD44 with SUMO Inhibitors in 8505C Cells The 8505C ATC cell line was tested for a response to SUMO inhibitors by western blot. **(A)** Cells were treated with PYR-41 at vehicle only (0), 2 μM (2) or 5 μM (5) as indicated. Western blot (top panel) showed reduction of CD44 expression with loss of SUMO-conjugated TFAP2A. FACS analysis (bottom panel) confirmed dose-dependent reduction of cell surface expression of CD44; numbers in colored boxes reports percent CD44-positive cells at each drug concentration and corresponds to flow diagram of the same color. **(B)** Experiments parallel to A demonstrate dose-dependent reduction of CD44 with AA at vehicle only (0), 10 μM (10), 25 μM (25) or 50 μM (50) as indicated by western blot (top panel) showing repression of CD44 and loss of SUMO-conjugated TFAP2A; bottom panel shows similar dose-dependent repression of cell surface expression of CD44 with AA; numbers in colored boxes reports percent CD44-positive cells at each drug concentration.

The effects of SUMO inhibition were examined in greater detail in 8505C cells. As determined by western blot, PYR-41 and AA treatment resulted in a dose-dependent repression of CD44 protein, which was accompanied by a loss of SUMO-conjugated TFAP2A (Figures 5A & 5B, top panels). FACS analysis was used to confirm the effect of SUMO inhibitors on the cell surface expression of CD44. The baseline expression in 8505C cells demonstrated that 79% of the cell population was CD44-positive (Figure 5A, bottom panel). PYR-41 at 2 and 5 μM reduced CD44-positive cells to 54% and 15%, respectively. The dose-dependent decrease in CD44 expression mimicked the western blot data. Similarly, we noted a dose-dependent decrease in cell surface expression of CD44 with AA treatment. At 50 μm AA, only 6% of the cells remained CD44-positive. The dose dependent response to AA mirrors the findings on western blot.

### Repression of CD44 with SUMO inhibition is mediated by TFAP2A

**Figure 6:**
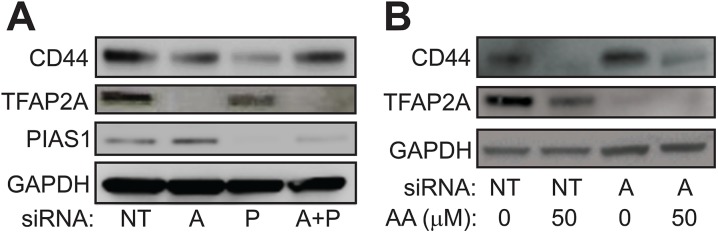
Effect of SUMO Inhibition are Mediated by TFAP2A **(A)** Western blot of 8505C cells transfected with non-targeting (NT), TFAP2A (A), PIAS1 (P) or combination of TFAP2A and PIAS1 (A+P) siRNA and analyzed for expression of CD44, TFAP2A, PIAS1 or GAPDH as indicated. Relative CD44 protein was NT: 1.0; A: 0.52; P: 0.34; A+P: 0.94. Relative TFAP2A protein was NT: 1.0; A: 0.003; P: 0.51; A+P: 0.02. Relative PIAS1 protein was NT: 1.0; A: 0.93; P: 0.12; A+P: 0.59. **(B)** Western blot of CD44 expression with vehicle (0) or anacardic acid (AA) at 50 μM (50) transfected with either non-targeting (NT) or TFAP2A (A) siRNA. Relative CD44 protein was NT/0: 1.0; NT/50: 0.13; AA/0: 1.0; AA/50: 0.44. Relative TFAP2A protein was NT/0: 1.0; NT/50: 0.35; A/0: <0.01; A/50: <0.01.

**Figure 7:**
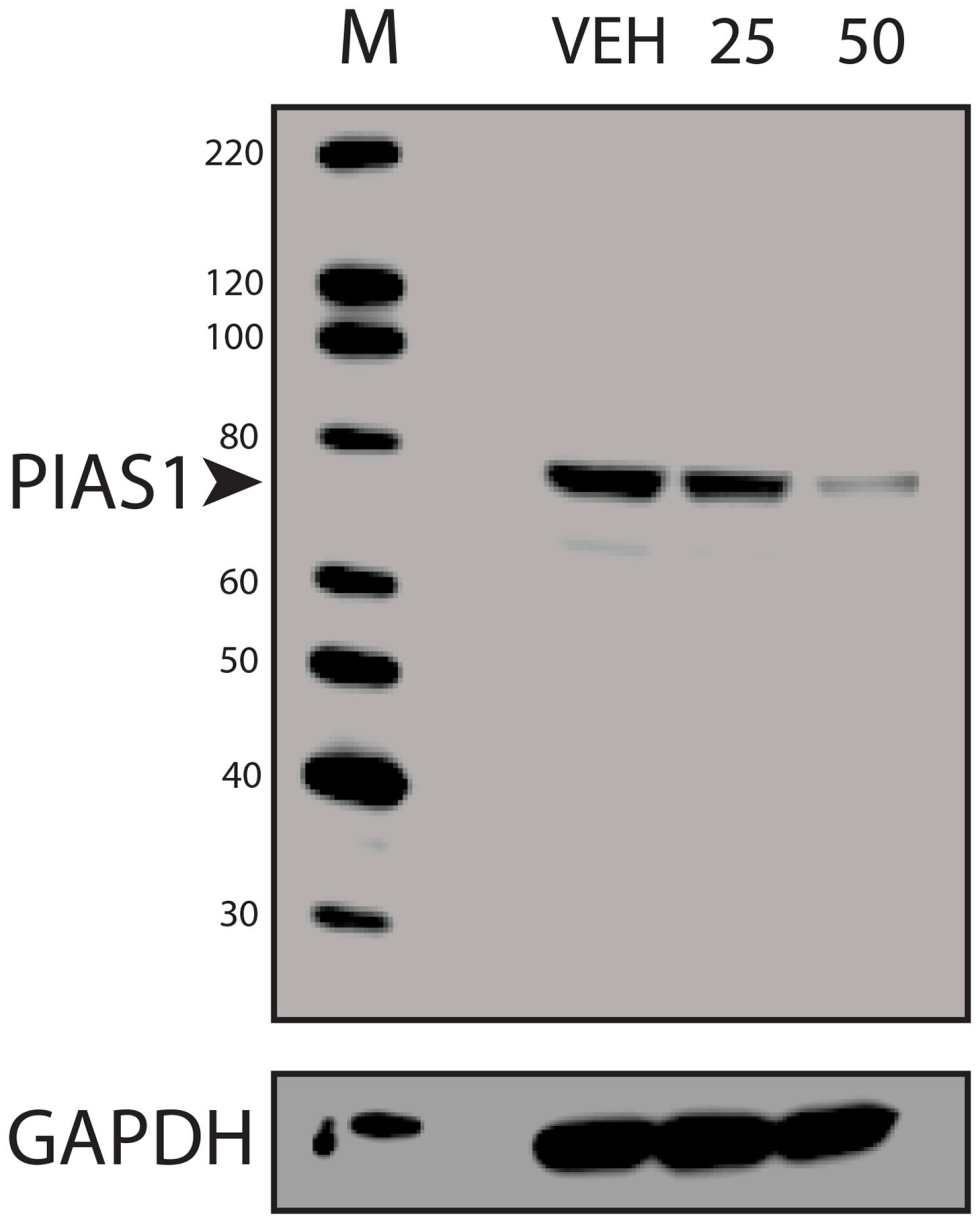
Repression of PIAS1 with Anacardic Acid Treatment Western blots of protein from 8505C cells treated with vehicle only (VEH) or AA at 25 μM (25) or 50 μM (50) for 96 hours and analyzed for PIAS1 expression with GAPDH loading control; M shows lane with markers. Data demonstrate a dose-dependent repression of PIAS1.

Previous studies in breast and colon cancer indicated that the effect of SUMO inhibition on CD44 expression was dependent upon TFAP2A [10, 13]. As noted above, response to SUMO inhibition correlated with expression of SUMO-conjugated TFAP2A and repression of CD44 was associated with loss of SUMO-conjugated TFAP2A. To confirm a functional role for TFAP2A on CD44 repression with SUMO inhibition, we examined the effect of SUMO inhibition with knockdown of TFAP2A. As seen in Figure 6A, knockdown of TFAP2A had minimal effect on CD44 expression, whereas, knockdown of PIAS1 significantly repressed CD44. However, knockdown of TFAP2A rescued repression of CD44 by PIAS1 knockdown (Figure 6A). Similarly, we tested the ability for AA to repress CD44 with knockdown of TFAP2A. With transfection of NT siRNA, AA repressed CD44 (Figure 6B); however, repression of CD44 by AA was abrogated by knockdown of TFAP2A. These findings indicated that repression of CD44 by SUMO inhibition was dependent upon SUMO conjugated TFAP2A in 8505C ATC cells. The results are in agreement with experiments in colon cancer cell lines showing that the SUMO resistant mutant K10R repressed CD44 expression [13]. Furthermore, we have noticed that AA treatment demonstrated a more robust effect on CD44 repression than knockdown of PIAS1. AA is known to block formation of the E1-SUMO intermediate [17]. We examined the effect of AA on PIAS1 expression. As seen in Figure 7, AA demonstrated a dose-dependent repression of PIAS1 expression. This finding indicates that AA represses the SUMO pathway through several mechanisms, including direct E1 SUMO inhibition and repression of PIAS1 E3 SUMO expression.

### SUMO inhibitors repress outgrowth of ATC xenografts

**Figure 8:**
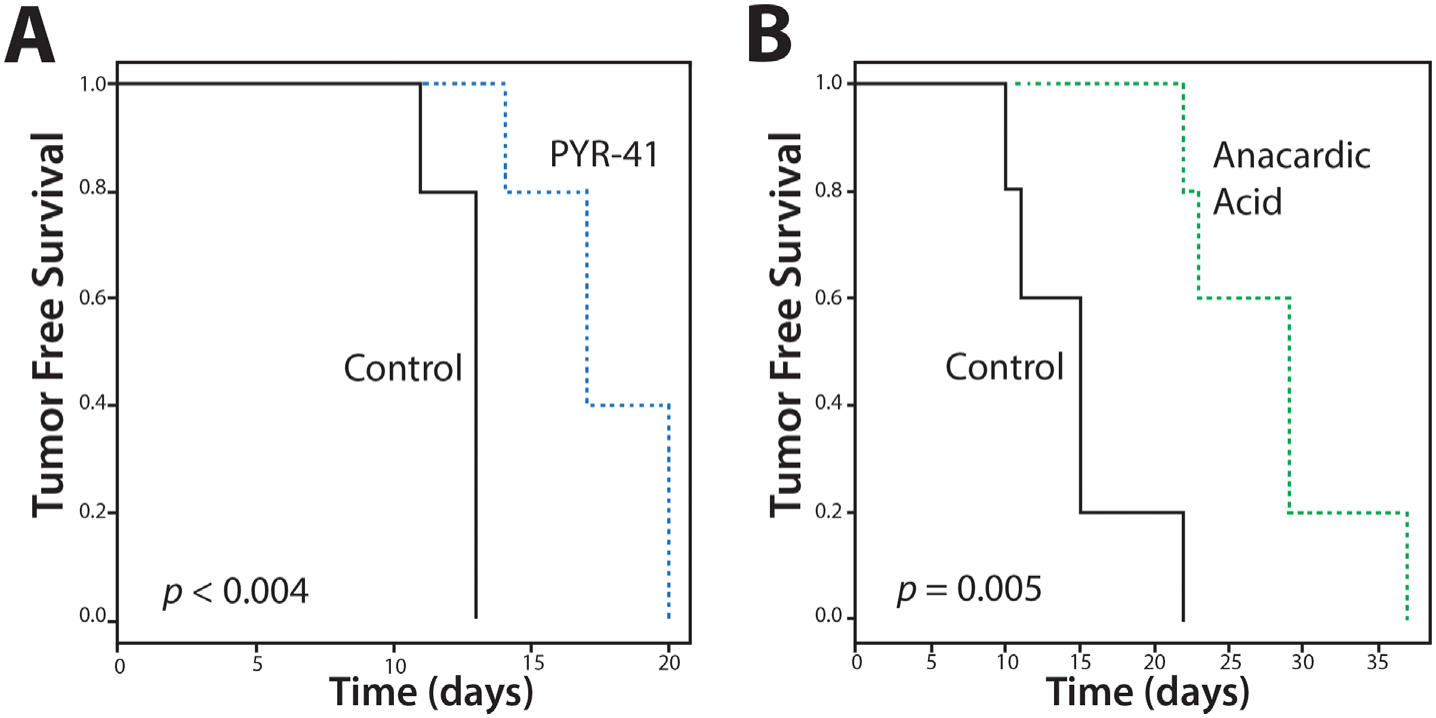
Tumor-free Survival (TFS) of Mice with SUMO Inhibitors Xenografts were inoculated into mice (n=5 per group) and treated with vehicle (control) or PYR-41 **(A)** or anacardic acid **(B)** and examined for tumor growth. Data demonstrates delay in TFS with SUMO inhibitors.

**Figure 9:**
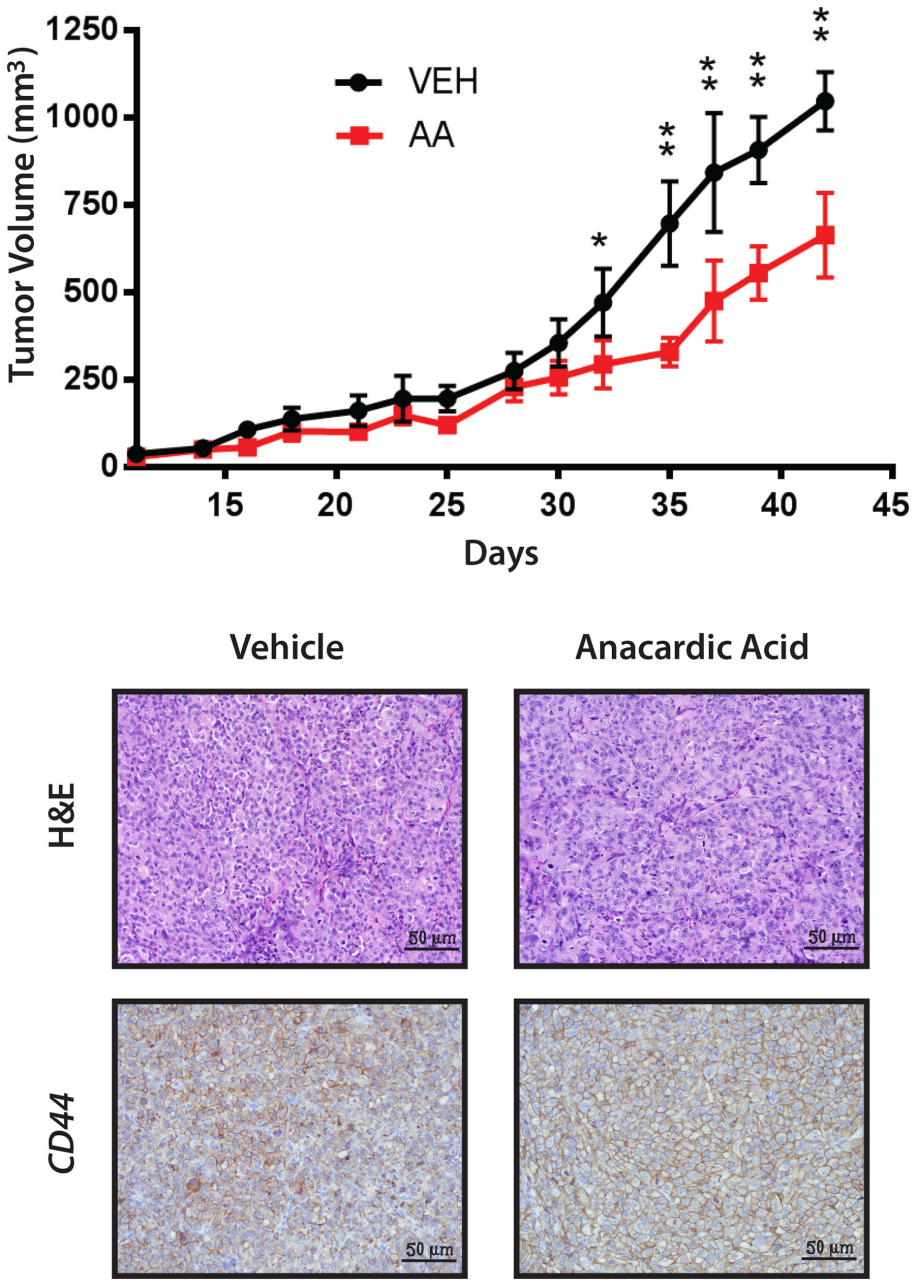
Xenografts of 8505C Analyzed for Growth, H&E and CD44 Mice with 8505C xenografts were gavaged with vehicle (VEH) or anacardic acid (AA) and evaluated for total volume of xenografts show a significant reduction in growth rate with AA treatment. ^*^<0.05, ^**^<0.001. Bottom panels show H&E (x200) and immunohistochemistry for CD44 of tumors from vehicle and AA treated animals, as indicated.

We considered whether SUMO inhibition induced differentiation of ATC; however, treatment with AA did not increase expression of genes associated with thyroid differentiation including *NKX2-1*, *SLC5A5/NIS* or *TG* (data not shown). On the other hand, the significant reduction of CD44-positive cells with SUMO inhibitors suggests an effect on the CSC/TIC population. Hence, we tested the effect of SUMO inhibitors on the outgrowth of 8505C tumor xenografts. Mice were inoculated with 8505C cells and randomly assigned to groups treated with PYR-41 vs. vehicle control. Control mice developed palpable tumors in a median 13 days compared to an extended 17 days for the PYR-41 treated group (p < 0.004) (Figure 8A). Parallel experiments were performed in mice treated with AA given by oral gavage compared to vehicle control gavage. Control mice developed tumors at a median 15 days compared to a median 29 days compared to the treatment cohort (p = 0.005) (Figure 8B). In a separate set of xenograft experiments, mice were flank injected with 8505C cells, gavaged with AA vs. vehicle, and tumor size was measured. As seen in Figure 9, AA treated animals developed significantly smaller tumors with a reduced growth rate noted after day 32. H&E staining of the tumors is shown in Figure 9, lower panel, and although tumors were smaller, they appeared to be identical histologically. We previously demonstrated that basal breast cancer xenografts developing in AA treated mice had a significant reduction in the CSC/TIC subpopulation as determined by FACS analysis [13]. Immunohistochemistry with CD44 was used to examine tumors from vehicle and AA treated animals. Tumors from both sets of animals demonstrated >75% membrane staining for CD44 and failed to clearly demonstrate a reduction in CD44 expression in tumors from AA treated animals (Figure 9, lower panel); the inability to see differences in CD44 likely indicates that IHC was not sensitive enough to demonstrate the effect on CD44 expression. However, the findings on balance are consistent with SUMO inhibitors reducing the CSC/TIC population in 8505C cells.

### TFAP2A expression in anaplastic thyroid cancer

**Figure 10:**
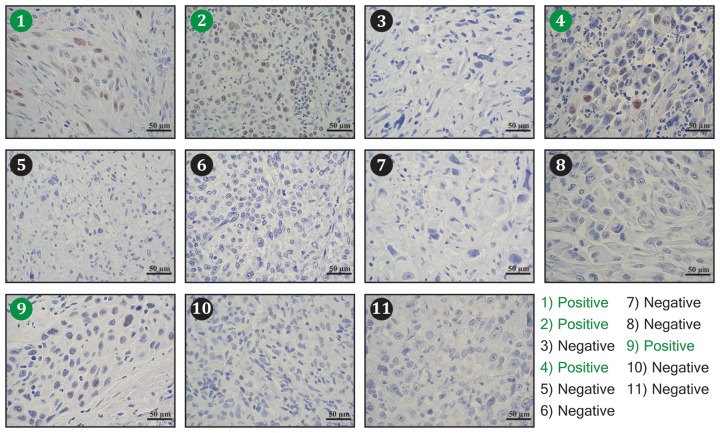
Immunohistochemistry for TFAP2A Expression in ATC Tumors Eleven archival ATC tumor specimens were examined for TFAP2A expression by immunohistochemistry. Tumors 1, 2, 4, and 9 (denoted in green) demonstrated positive brown nuclear TFAP2A staining.

**Figure 11:**
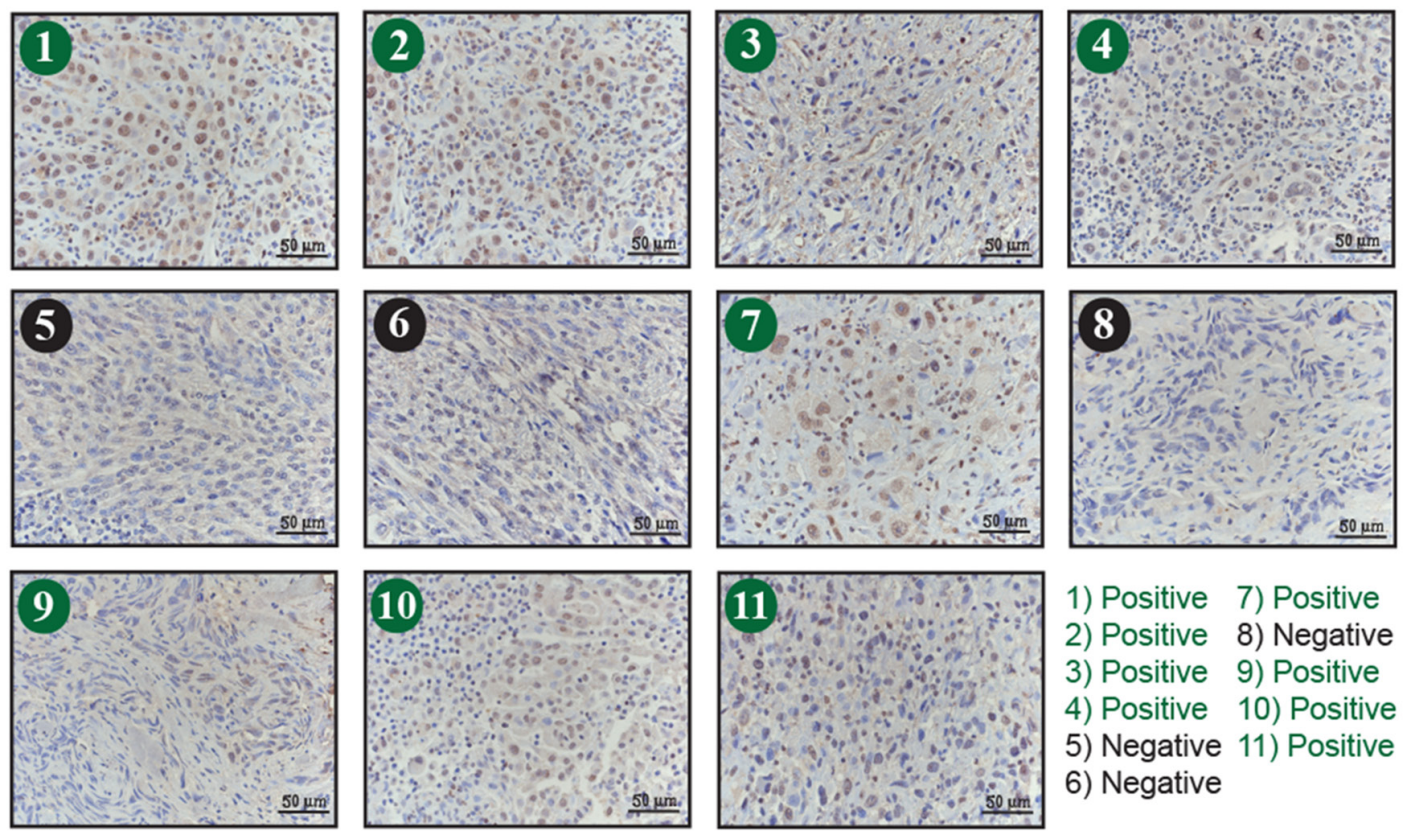
Immunohistochemistry for PIAS1 Expression in ATC Tumors Eleven archival ATC tumor specimens (same as in Figure 10) were examined for PIAS1 expression by immunohistochemistry. Tumors 1, 2, 3, 4, 7, 9 10 and 11 (denoted in green) demonstrated positive brown PIAS1 staining.

The findings suggest that TFAP2A plays an important role in mediating the effects of SUMO inhibitors in ATC. However, little is known about the expression of TFAP2A in primary ATC. With IRB approval, eleven archival blocks of ATC were retrieved and assessed for TFAP2A expression by immunohistochemistry. TFAP2A expression was identified in 4 (36%) of the 11 tumors (Figure 10). In all cases, the TFAP2A expression was nuclear. The tumors were also assessed for CD44 and PIAS1 expression. All tumors were strongly positive for CD44 by IHC (data not shown). PIAS1 expression varied from 0 to 90% (Figure 11). Interestingly, there was a trend for an association between expression of TFAP2A and PIAS1; all TFAP2A-positive tumors were also PIAS1-positive, whereas, only 4 of the 7 TFAP2A-negative tumors were PIAS1-positive (p=0.23).

## DISCUSSION

**Figure 12:**
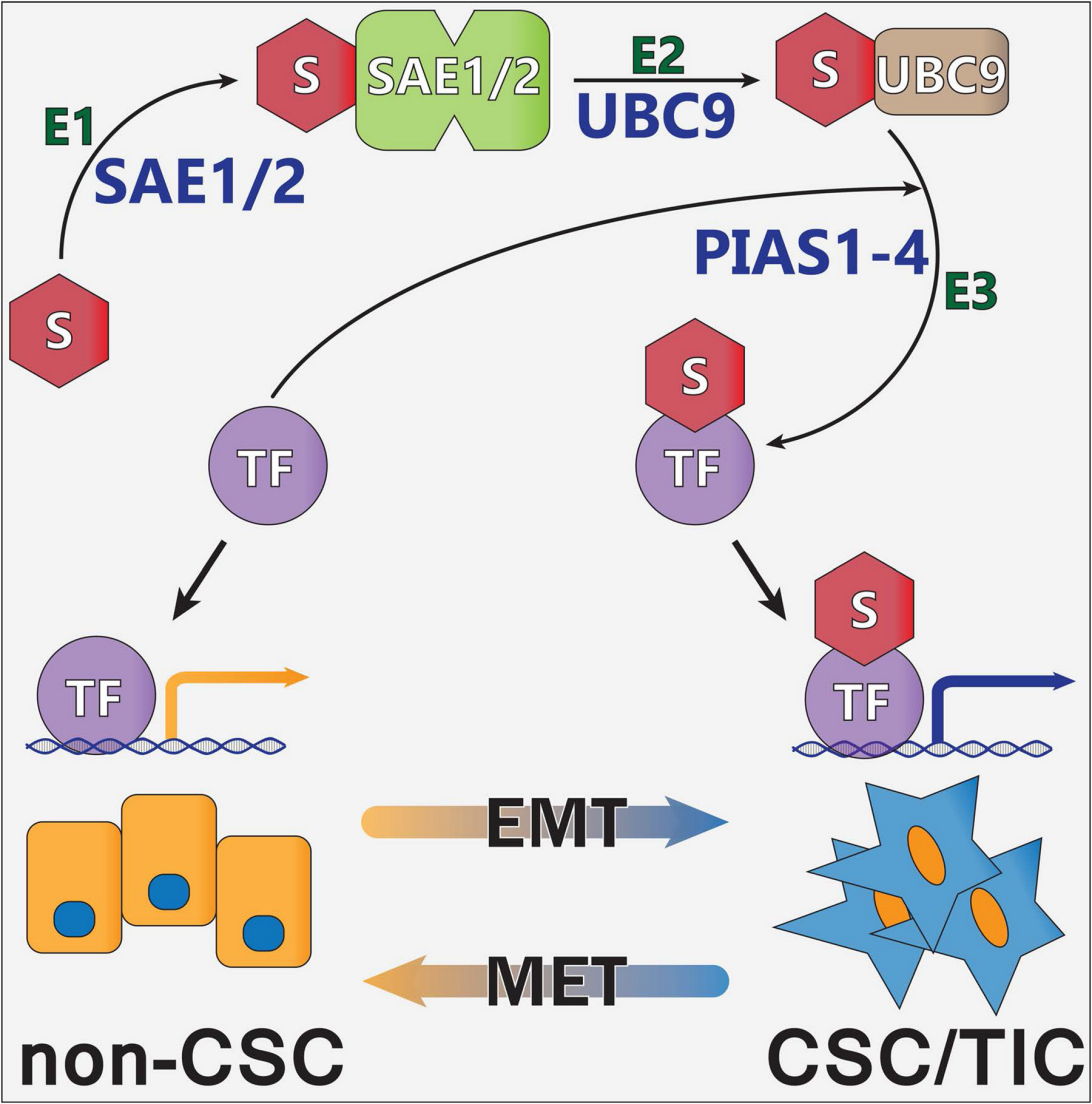
Model of Regulation of CSC/TIC Phenotype Through Sumoylation of TFAP2A Shown if the proposed model of SUMO conjugation of TFAP2A (TF) controlling patterns of gene expression. SUMO unconjugated TFAP2A is sumoylated through an enzymatic cascade involving E1 SUMO enzymes SAE1/2, E2 SUMO enzyme UBC9 and E3 SUMO ligases (including the PIAS family, PIAS1-4) resulting in SUMO conjugation of target TFAP2A (TF). SUMO unconjugated TFAP2A regulates expression of genes associated with the differentiated non-CSC subtype. SUMO conjugated TFAP2A regulates the pattern of gene expression associated with the CSC/TIC subtype. The model demonstrates the plasticity of CSC/TIC altered through SUMO conjugation of the TFAP2A transcription factor.

Earlier studies in breast and colon cancer demonstrated that inhibiting the SUMO pathway reduced the outgrowth of tumor xenografts, which appeared to be due to a significant reduction in the CSC/TIC population [10–13]. The current studies indicate that similar SUMO-dependent mechanisms are functional in a subset of ATC tumors. The effect of SUMO inhibition on the CSC/TIC subpopulation was dependent, at least in part, on SUMO-conjugated TFAP2A and our findings in ATC further implicate a role for TFAP2A in mediating the repression of CD44 and the CSC/TIC phenotype. Whereas in breast and colon cancer models we had evidence that SUMO inhibition affected the CSC/TIC phenotype in the majority of tumors tested, the effect of SUMO inhibition was identified in a minority of ATC cell lines. That the response was associated with the expression of SUMO-conjugated TFAP2A and knockdown of TFAP2A abrogated the response in 8505C cells lends further support for implicating SUMO-conjugated TFAP2A in the response to SUMO inhibition. Based on these findings, we have developed a working model of how SUMO conjugation of the TFAP2A transcription factor alters patterns of gene expression associated with the transition of cancer cells from the more differentiated phenotype to the CSC/TIC subtype (Figure 12). Further studies are needed to clarify the mechanism whereby the post-translational modification by SUMO conjugation of TFAP2A alters its transcriptional activity and consequent pattern of gene expression.

Although ATC accounts for only 1-2% of all cases of thyroid cancer, it is the cause of 30-50% of all thyroid cancer specific deaths [18, 19]. The five-year cancer specific survival (CSS) for ATC is only 7-10% with a median survival of 3-5 months [20, 21]. Patients with ATC who present with distant metastases have a 2-year survival rate of only 2% [22]. Multimodal therapy including surgical resection, chemotherapy and radiation has been used to gain local control but has had minimal impact on patient survival [22] with some studies concluding that aggressive multimodality treatment of patients with ATC has failed to have any impact on survival [21, 23]. There is a need for a better understanding of the biology of ATC, which may lead to novel therapeutic approaches that could improve survival of patients with this aggressive cancer [24]. The findings in the current study suggest that targeting the SUMO pathway may offer a novel approach to treating patients with ATC. The effects of SUMO inhibition appear to be mediated through TFAP2A and as many as one-third of ATC tumors may respond to this novel approach to therapy. Previous studies reported that TFAP2A was upregulated in thyroid cancer compared to normal thyroid [25]. Databases such as TCGA report that 1-2% of thyroid tumors display overexpression of TFAP2A, though there does not appear to be any obvious differences in survival for this group. To our knowledge, no previous data has been reported for TFAP2A expression in ATC.

The undifferentiated thyroid cancer phenotype of ATC is characterized by a loss of expression of differentiation markers such as thyroglobulin and the sodium-iodide symporter (*NIS*) as well as an increased proliferative rate. Evidence from histopathological and molecular analysis indicates that ATC often develops from well-differentiated papillary and follicular thyroid cancer through a process of de-differentiation [26]. Whereas most well differentiated thyroid cancers have a normal *TP53* gene, most ATC tumors harbor mutations of p53 commonly targeting codon 273 [27]. Studies have also indicated a cooperative effect on transcription through interactions between TFAP2A and p53 [28, 29]. Recurrent mutations in *BRAF* and *NRAS* have been identified in ATC, which are mutually exclusive with ATC cell lines having either mutated *BRAF* and *TP53* or *NRAS* and *TP53* genotypes [30]. Components of the Notch signaling pathway are reduced in ATC compared to well-differentiated thyroid cancers. In addition, forced over expression of Notch-1 in thyroid cells induced thyroid cell differentiation including near-normal expression of the *NIS* and thyroperoxidase (*TPO*) genes and a reduction in the proliferation rate.

In addition to markers associated with the undifferentiated phenotype, a comparison of ATC to well differentiated carcinomas also demonstrated an increase in an epithelial-mesenchymal transition (EMT) signature [31]. By immunohistochemistry several transcription factors implicated in EMT induction, such as SLUG (*SNAI2*), *TWIST1* and *ZEB1* are all up-regulated in ATC compared to normal thyroid and well-differentiated thyroid cancers and coordinately act to repress E-cadherin (*CDH1*) expression [32–34]. In addition, knockdown of TWIST1 using siRNA in ATC cell lines induced apoptosis and inhibited cell migration and invasion; alternatively, overexpression of TWIST1 by transfection into thyroid carcinoma cell lines induced resistance to apoptosis and increased cell migration and invasion [34]. Previous studies have demonstrated that loss of TFAP2C expression induced EMT in luminal breast cancers [9] and recent findings confirm a role of TFAP2C in regulating EMT in lung cancer [35]; furthermore, inhibiting sumoylation of TFAP2A activated a transcriptional program replicating TFAP2C functionality that induced mesenchymal-epithelial transition (MET) in basal breast cancer [10]. The current studies demonstrate that similar SUMO-sensitive, TFAP2A-dependent mechanisms regulating EMT/MET are active in a subset of ATC cell lines (Figure 12).

In basal breast cancer, a close relationship has been established between expression of the EMT signature and the cancer stem cell (CSC) or tumor-initiating cell (TIC) [36]. The CSC population in breast and several other cancer types are defined by the expression of certain cell surface markers including CD44, CD133, ALDH1 and ESA [3, 37, 38]. Similar studies have demonstrated an association between the EMT signature and the CSC population in ATC. There is an expansion of the CSC population in ATC compared to well-differentiated thyroid cancers with intense expression of the markers CD133, CD44 and nestin [14, 39]. However, the role of nestin as a marker for the CSC population in ATC is an area of debate [40]. Expansion of the CSC population in ATC may account for the aggressive clinical course, propensity for metastasis and poor response to conventional chemotherapy. Functional analysis has demonstrated an important role for the sonic hedgehog (Shh) pathway signaling through Snail in maintaining CSC self-renewal in ATC [41]. Todaro et. al. [42] isolated thyroid CSCs using the marker ALDH1 and showed that ATC have an expansion of the CSC population compared to well-differentiated thyroid tumors. ALDH1^high^ CSCs isolated from ATC have enhanced proliferation, invasiveness and metastatic potential. Silencing Akt or Met in CSCs isolated from ATC dramatically reduced tumorigenesis and completely abrogated metastatic propensity. The current study provides additional support for SUMO-unconjugated TFAP2A repressing expression of CD44 and thereby inhibiting the CSC population in ATC. The use of SUMO inhibitors to specifically eliminate the CSC population in ATC may offer a novel approach that may lead to a more robust response to conventional chemotherapy at least in a proportion of ATC tumors.

## MATERIALS AND METHODS

### Cell culture

The KAT-18 cell line was obtained from Dr. Kenneth Ain (The University of Kentucky), the 8505C cells were a kind gift from Dr. Sareh Parangi (Harvard Medical School, Boston, MA, USA) and the Uhth74-c17 (herein referred to as Uhth74) and SW-1736 cell lines were obtained from Dr. Nils-Erik Heldin (Uppsala University, Uppsala). Uhth74 and 8505C cell lines were maintained in DMEM and KAT-18 and SW-1736 were maintained in RPMI media in a standard humidified incubator at 37°C and 5.0% carbon dioxide. All media was supplemented with 10% FBS, 1% 100X Pen/Strep antibiotics, and 1% 100X GlutaMAX (all components from Life Technologies, Madison, WI, USA).

### siRNA transfections

Small interfering RNAs were obtained for TFAP2A, PIAS1, and non-targeting (NT) (Ambion Silencer Select, Carlsbad, CA, USA). Transfections were performed for 96 hours using Lipofectamine RNAImax reagent (Invitrogen, Carlsbad, CA, USA) according to manufacturer protocol.

### *In vitro* treatment with SUMO inhibitors

Cells were plated in 6-well plates and treated with varying concentrations of the SUMO inhibitors anacardic acid (Tocris, Bristol, UK) and PYR-41for 72-96 hours and were then collected for RNA, protein or FACS analysis. Stock solutions were used within 6 weeks.

### Real-time PCR

Total RNA was isolated 96 hours after siRNA transfection and 72-96 hours after treatment with SUMO inhibitor using the RNeasy Mini kit (Qiagen, Hilden, Germany). RNA was converted to cDNA using the High Capacity cDNA Reverse Transcription Kit (Applied Biosystems, Foster City, CA, USA). Quantitative PCR was performed in quadruplicates to determine relative gene expression using TaqMan primer/probe combinations for TFAP2A, PIAS1 and CD44 with 18s rRNA for the endogenous control (Applied Biosystems).

### Western blot analysis

Total protein was isolated 96 hours after siRNA transfection and 72-96 hours after SUMO inhibitor treatment using RIPA buffer with Halt Protease Inhibitor Cocktail (100X) (ThermoFisher Scientific, Rockford, IL, USA). Antibodies for TFAP2A (AbCam, Cambridge, MA, USA), PIAS1 (AbCam), CD44 (RD Systems, Minneapolis, MN, USA), Sumo 1 (AbCam), Sumo 2/3 (AbCam) and GAPDH (Santa Cruz Biotechnologies, Santa Cruz, CA, USA) were used for Western blot analysis. Immunoprecipitations were performed using the Pierce Co-Immunoprecipitation Kit (Thermo Fisher) using antibodies for TFAP2A (AbCam) or Sumo 1 (AbCam) and Sumo 2/3 (AbCam) with rabbit IgG as a control.

### Flow cytometry

8505C cells were detached using trypsin, washed with PBS, and incubated for 30 minutes with CD44-APC (RD Systems). Living cells were identified using Hoechst stain (Invitrogen). Flow cytometry was performed on a FACS DiVa Machine (Becton-Dickinson, San Jose, CA, USA).

### Tumor xenografts

Following University of Iowa IACUC approval, twenty female nu/J nude mice (Jackson Laboratory, Bar Harbor, ME, USA) were flank injected with 1×10^6^ 8505C cells suspended in media/Matrigel (BD Biosciences, San Jose, CA, USA) in a 1:1 ratio. Mice were then randomized to treatment with either anacardic acid (AA) (10mg/kg) or vehicle (10% DMSO) oral gavage five times a week. In a separate experiment 10 additional mice were likewise flank injected with 1×10^6^ 8505C cells suspended in media/Matrigel (1:1 ratio) and treated with either intraperitoneal (IP) PYR-41 (10mg/kg) every other day for 15 days or vehicle IP injection (10% DMSO in water). Mice were followed for tumor development. For experiments with AA gavage, tumor volume was calculated in mm^3^ using the width, length and height of the tumors as described [43]. Experimental protocols were approved by the University of Iowa Institutional Animal Care And Use Committee (IACUC). Animals were euthanized per IACUC protocol when tumors reached 2 cm. The investigative team monitored all mice daily and an independent veterinary team additionally monitored the mice daily for signs of undue suffering and illness. Animals were euthanized by CO_2_ asphyxiation and subsequent cervical dislocation. No mice experienced premature death and mice did not require use of analgesics and anesthetics.

### Immunohistochemistry

Tissue blocks for previously banked ATC specimens were pulled after being found to meet IRB exemption criteria. Samples were collected without regard for sex of the patient. Formalin fixed paraffin embedded tissue was cut onto glass slides, antigen unmasked with citrate buffer, pH 6.0 in a pressure cooker at 125°C for 5 minutes. After quenching with 3% H_2_O_2_ and washing, TFAP2A antibody (Santa Cruz Biotechnology, Dallas, TX, USA) was added at a 1:100 dilution for 60 minutes at room temperature. A horseradish peroxidase based visualizing system was utilized and the slides then stained with hematoxylin. This protocol was repeated using PIAS1 antibody (AbCam) at a 1:100 dilution on the same ATC specimens. Immunohistochemistry was performed on ATC tumors from patients and xenografts using a CD44 antibody (R&D Biosystems) at a 1:3000 dilution. TFAP2A, PIAS1 and CD44 expression was quantified by R.A.R. Staining of greater than 10% was considered positive.

### Statistical analysis

Statistical comparisons between quantitative PCR data was performed using the Applied Biosystems manufacturer’s software and is presented as 95% confidence intervals. Tumor-free survival curves were compared by logrank test using R (https://www.r-project.org/) and tumor volume was compared by two-way ANOVA.

## SUPPLEMENTARY MATERIALS FIGURE



## Abbreviations

AA: Anacardic acid
ATC: Anaplastic thyroid cancer
cDNA: Complimentary DNA
CSC: Cancer stem cells
EMT: Epithelial-mesenchymal transition
FACS: Fluorescence activated cell sorting
IP: Intraperitoneal
MET: Mesenchymal-epithelial transition
PCR: Polymerase chain reaction
siRNA: small interfering RNA
SUMO: Small ubiquitin-like modifier
TFAP2A: Transcription factor AP2-alpha
TIC: Tumor initiating cells.
