# Supplementary material for: Targeting the SUMO pathway as a novel treatment for anaplastic thyroid cancer
Source: Oncotarget. 2017 Oct 23;8(70):114801–15. doi: 10.18632/oncotarget.21954 (PMC5777733; doi:10.18632/oncotarget.21954)
Supplement: Supplementary file 1 [file oncotarget-08-114801-s001.pdf]

Targeting the SUMO pathway as a novel treatment for anaplastic thyroid cancer

SUPPLEMENTARY MATERIALS

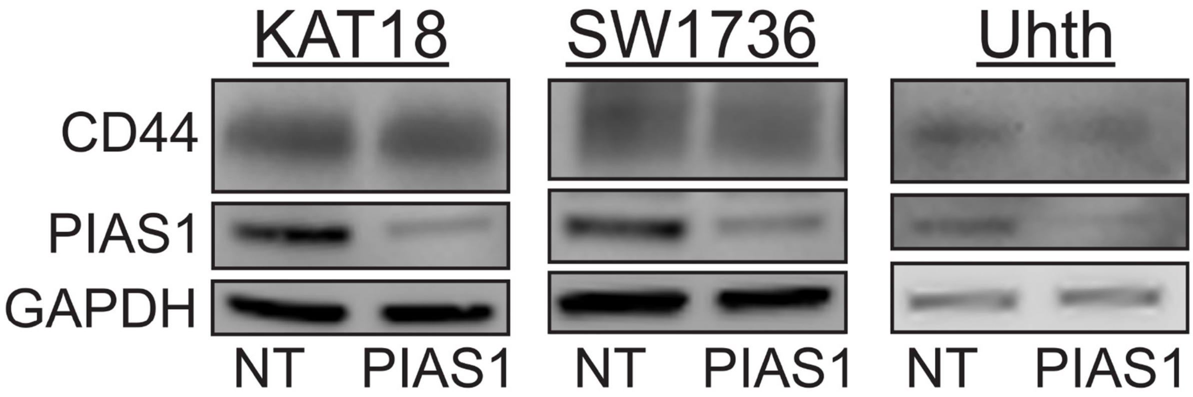

Supplementary Figure 1: Confirmation of Knockdown of PIAS1. Western blots showing knockdown of PIAS1 after transfection with siRNA to PIAS1 compared to non-targeting siRNA (NT) in cell lines shown. CD44 showed no change in expression and GAPDH confirmed equal loading of protein.
